# Publisher Correction: Dual roles of the sterol recognition region in Hedgehog protein modification

**DOI:** 10.1038/s42003-020-1023-0

**Published:** 2020-06-02

**Authors:** Rahul Purohit, Daniel S. Peng, Erika Vielmas, Alison E. Ondrus

**Affiliations:** Division of Chemistry and Chemical Engineering, California Institute of Technology, Pasadena, CA 91125 USA

**Keywords:** Chemical modification, Chemical tools

Correction to: *Communications Biology* 10.1038/s42003-020-0977-2, published online 21 May 2020.

In the original published version of the article, an error was introduced into Fig. 1a during the typesetting process. The error has been corrected in the PDF and HTML versions of the paper.

