# Supplementary material for: Dual roles of the sterol recognition region in Hedgehog protein modification
Source: Commun Biol. 2020 May 21;3:250. doi: 10.1038/s42003-020-0977-2 (PMC7242414; doi:10.1038/s42003-020-0977-2)
Supplement: Supplementary file 1 — Supplementary Information [file 42003_2020_977_MOESM1_ESM.pdf]

Supplementary Figures

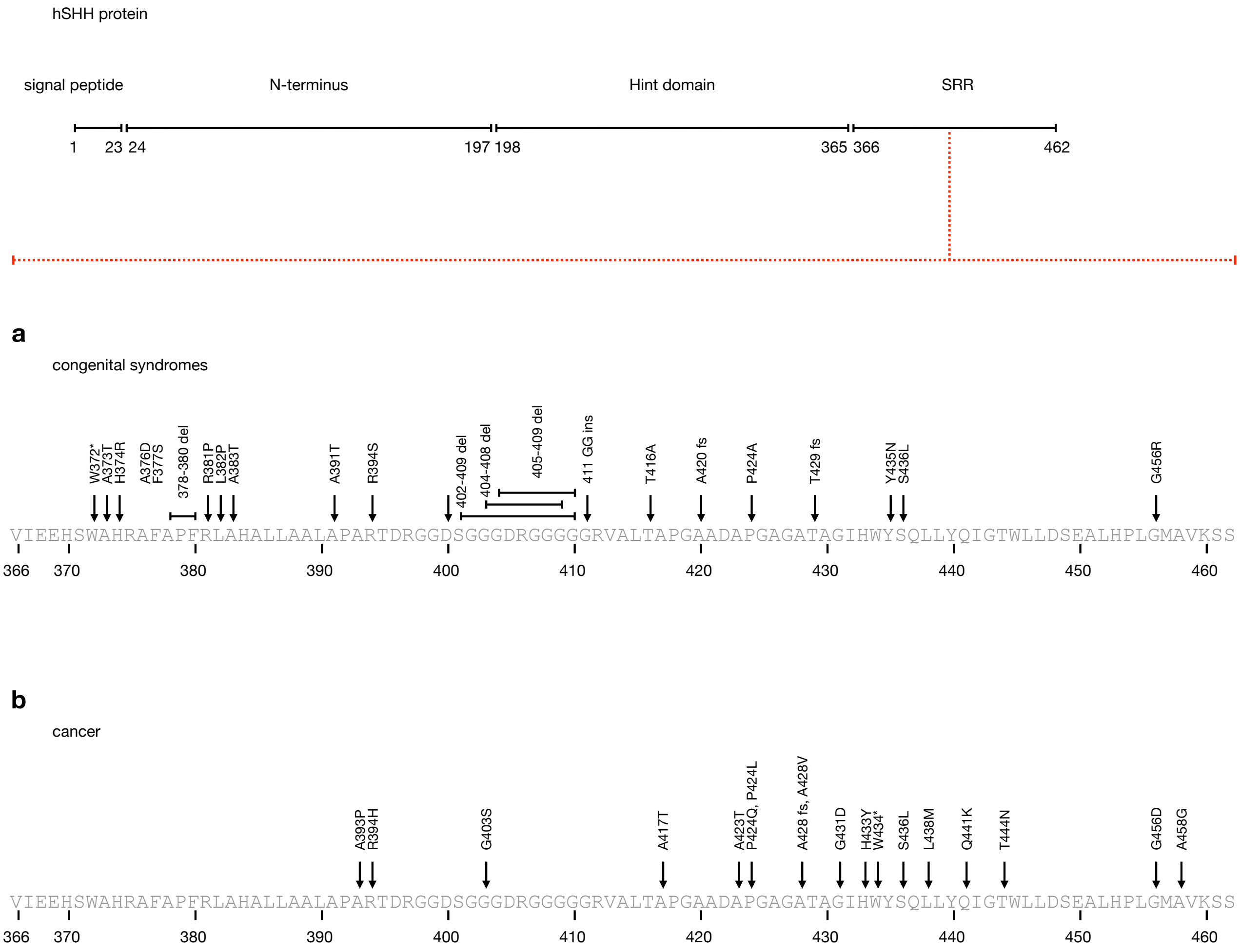

Supplementary Fig. 1 Disease-associated mutations in the hSHH SRR. **a** Mutations within the hSHH SRR associated with holoprosencephaly (Supplementary Refs. 1, 2, 3, 4, and 5) and dbSNP.[6] **b** Mutations within the hSHH SRR discovered in human cancer tissues. Data from COSMIC,[7] ClinVar[8], cBioPortal,[9] and dbSNP.[6]

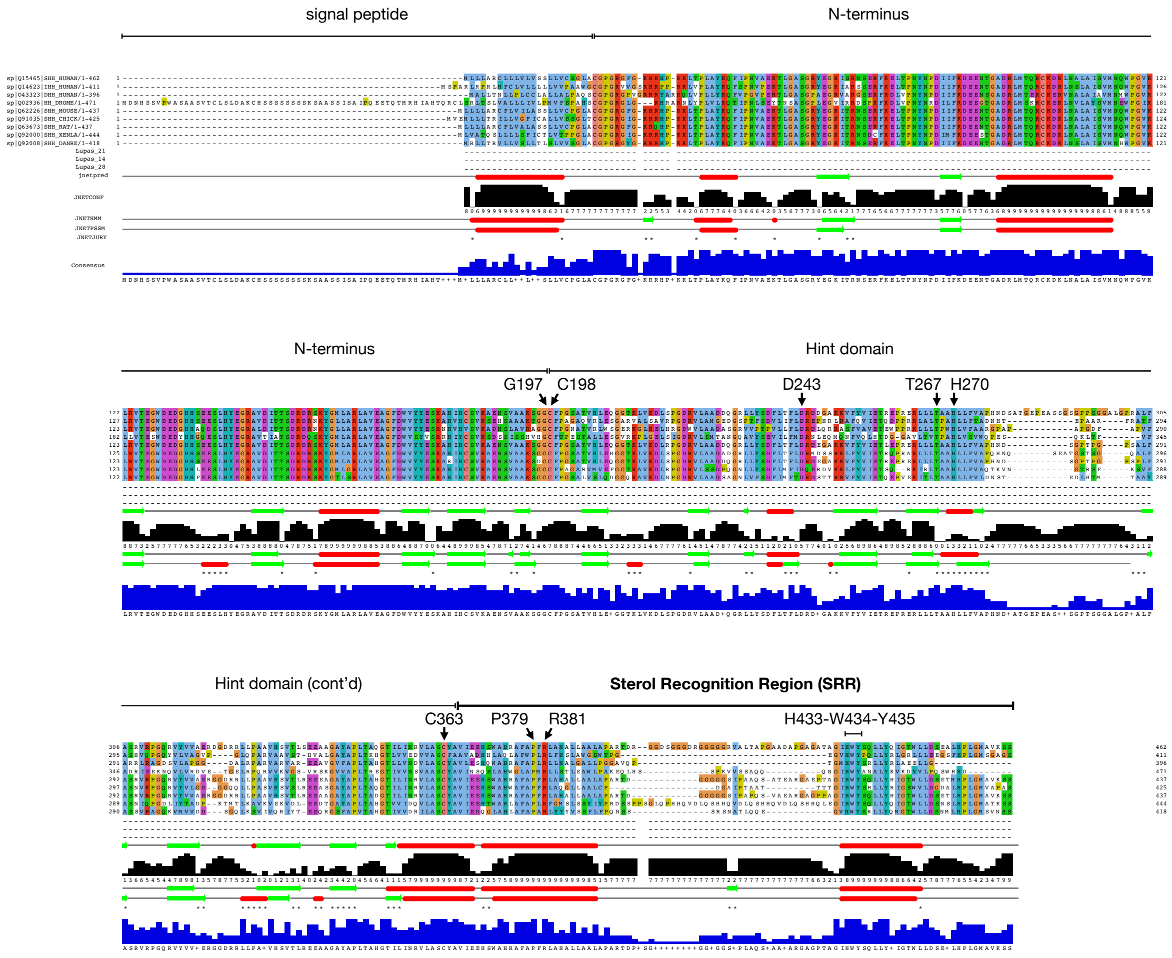

Supplementary Fig. 2 Alignment of selected Hh proteins. Clustal alignment showing conserved signal peptide, N-terminal domain, Hint domain, and SRR; Hint domain catalytic residues are indicated (hSHH numbering). Plot below shows Jpred4 secondary structure prediction for the consensus sequence.[10,11] Red cylinders indicate α-helices; green arrows indicate β-sheets; JNETCONF indicates confidence in secondary structure prediction at each residue.

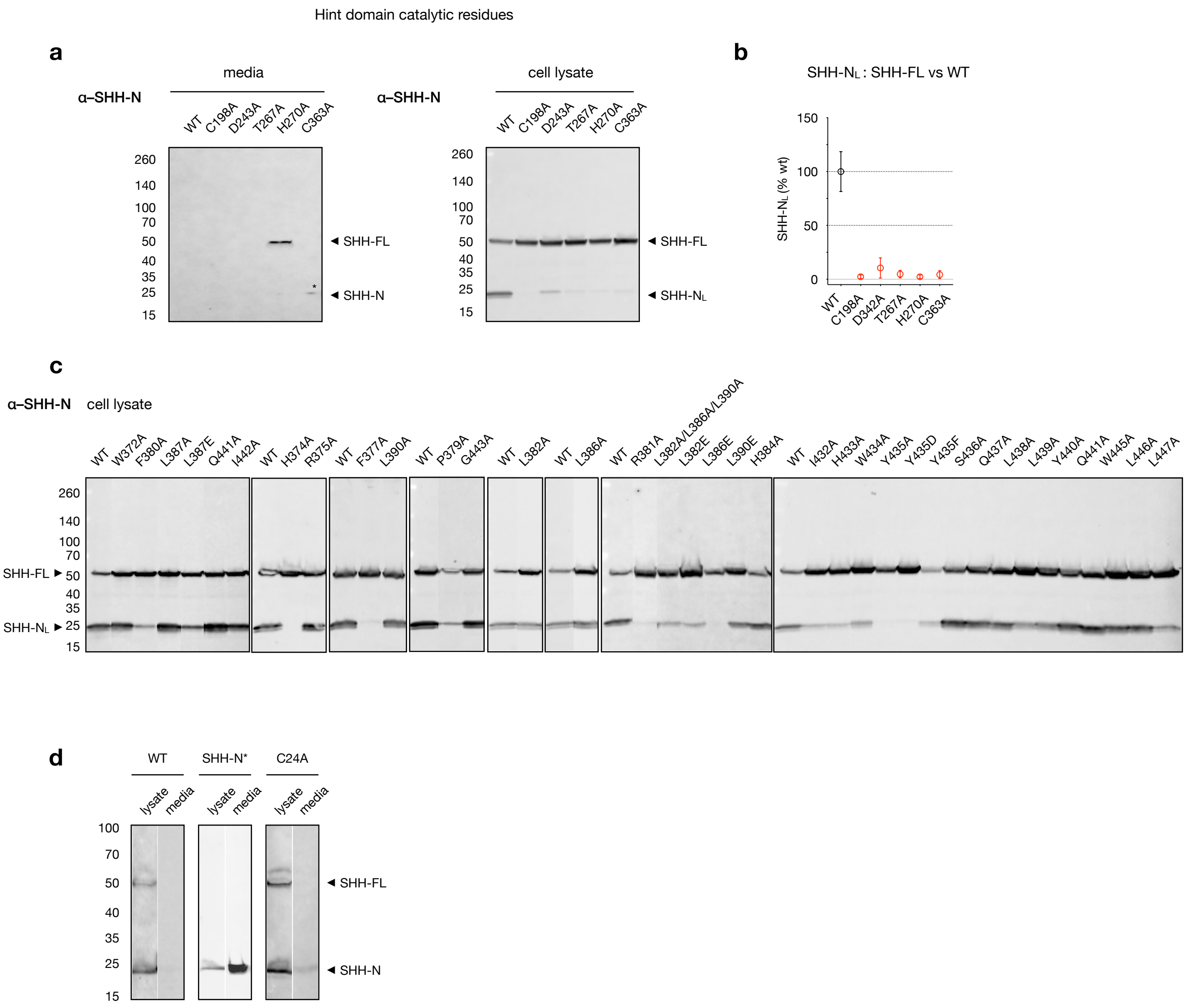

Supplementary Fig. 3 Western blot analysis of secreted and cell-associated protein from hSHH-overexpressing cells. **a** Western blot analysis of wild-type hSHH protein and Hint domain mutants expressed in HEK293T cells. Left: Analysis of hSHH-N produced by each mutant and precipitated from cell media. Right: Western blot of hSHH-N present in the corresponding lysates (hSHH-N_L) of each mutant. **b** Plot of relative hSHH-N_L production versus wild-type protein by each mutant for *n* = 3–10 biological replicates. A biological replicate for wild-type protein was analyzed in each blot. Symbols represent the mean production of hSHH-N_L versus wild-type protein for each mutant ± s.d. Mutants that produced $\leq$ 50% hSHH-N_L protein relative to wild-type protein are indicated in red. **c** Western Blot image for each mutant in Fig. 2b, 2c, 2e, and 2f. Relative hSHH-N_L production for each mutant was calculated from *n* = 3–10 biological replicates and plotted in Fig. 2. **d** Comparison of protein retained in lysates or secreted into cell media for wild-type protein, cholesteroylation-deficient hSHH-N (residues 1-197 of hSHHN, hSHH-N*), and a palmitoylation-deficient hSHH mutant (C24A).

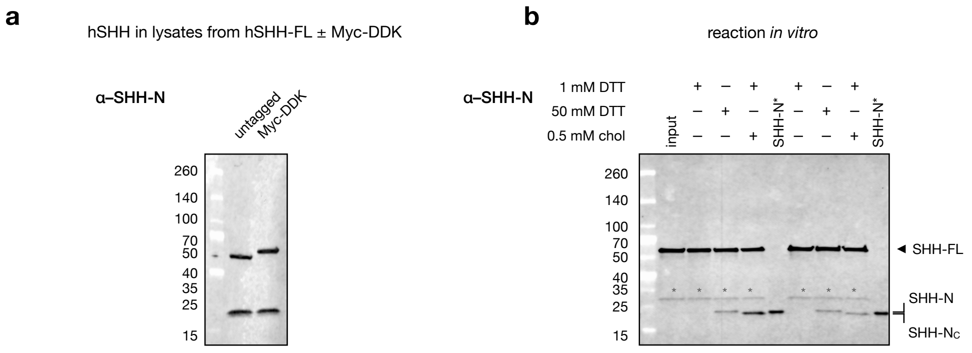

Supplementary Fig. 4 A C-terminal Myc-DDK tag enables isolation of hSHH-FL from overexpressing HEK293T cells and cholesterolysis *in vitro*. a Western blot of HEK293T cells transfected with untagged and C-terminal Myc-DDK tagged hSHH-FL shows equivalent production of cell-associated hSHH-N (hSHH-N_L). **b** Full blot showing *in vitro* cholesterolysis of wild-type hSHH protein. Stars (*) indicate residual mouse IgG from the agarose-conjugated anti-FLAG antibody.

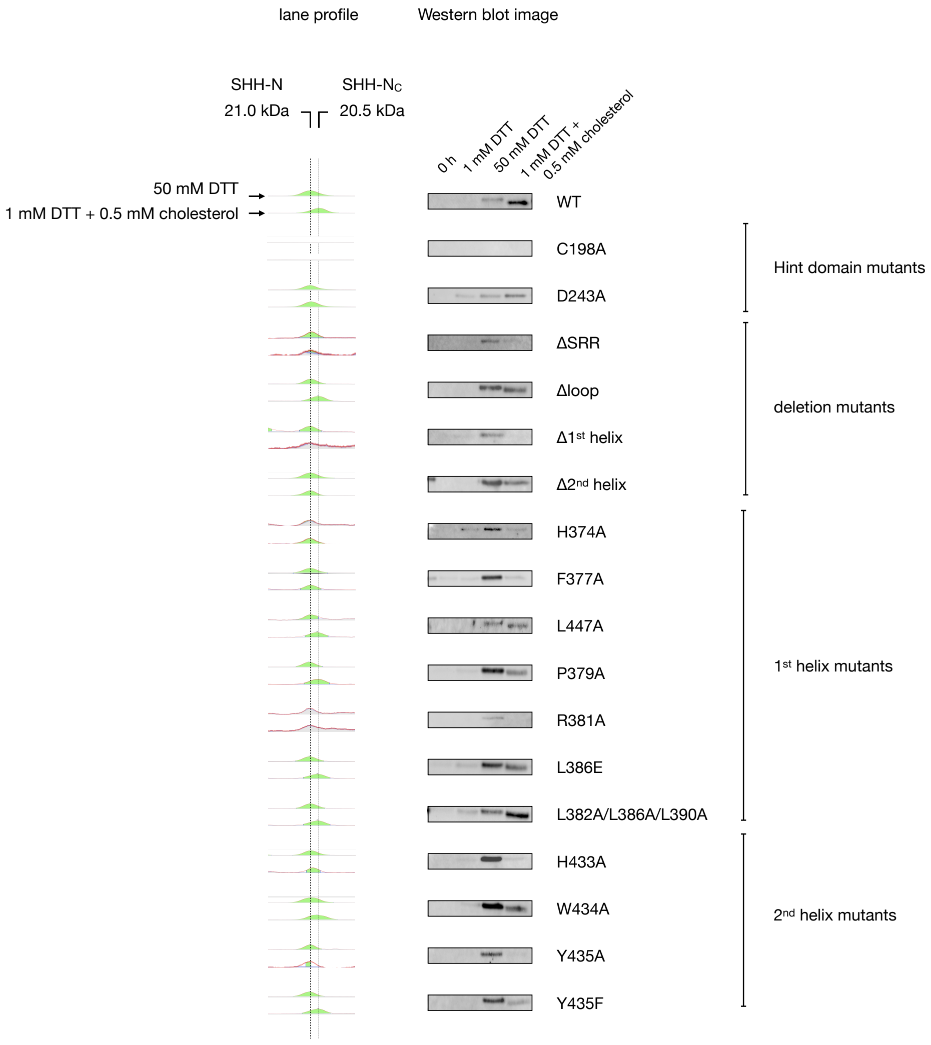

Supplementary Fig. 5 Determination of cholesterolysis *in vitro* by electrophoretic mobility shift of hSHH-N$_C$. Traces to the left show lane profiles with electrophoretic mobilities standardized to calibrated protein markers using Image Lab software. Panels to the right show corresponding Western blot images.

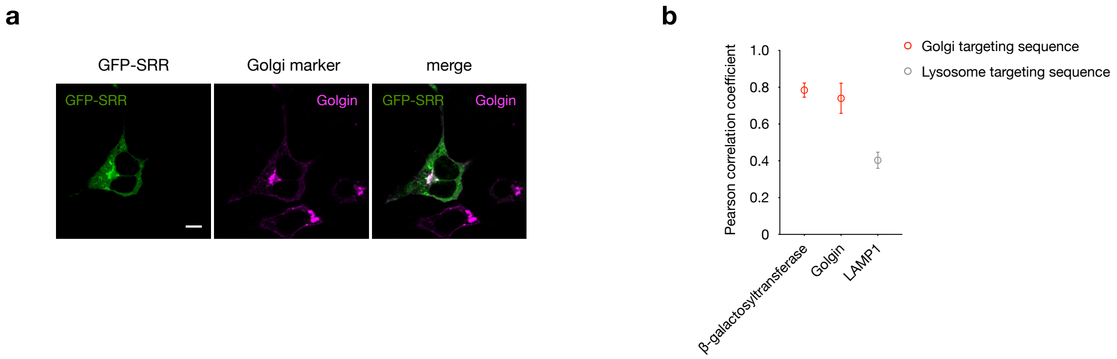

Supplementary Fig. 6 Subcellular colocalization studies with EGFP-hSHH(SRR). **a** Confocal microscopy image of HEK293T cells co-transfected with EGFP-SRR (EGFP-hSHH(365-462)) and mCherry fused to the Golgi-targeting sequence of Golgin. **b** Plot of Pearson correlation analysis of overlap between mCherry markers fused to Golgi-targeting sequences from β4-galactosyltransferase-1 (β4Gal-T1) and Golgin, and lysosome-targeting sequence from LAMP1. Symbols represent the mean value of Pearson correlation coefficient ± s.d. from n = 3–10 cells in three separate experiments, calculated using the Coloc 2 plugin in ImageJ. Scale bar = 10 µm.

Supplementary References

[1] Roessler, E. et al. The mutational spectrum of holoprosencephaly-associated changes within the SHH gene in humans predicts loss-of-function through either key structural alterations of the ligand or its altered synthesis. *Hum. Mutat.* **30**, E921-35 (2009).

[2] Hehr, U. et al. Wide phenotypic variability in families with holoprosencephaly and a sonic hedgehog mutation. *Eur. J. Pediat.* **163**, 347–352 (2004).

[3] Dubourg, C. et al. Molecular screening of SHH, ZIC2, SIX3, and TGIF genes in patients with features of holoprosencephaly spectrum: Mutation review and genotype–phenotype correlations. *Hum. Mut.* **24**, 43–51 (2004).

[4] Roessler, E. et al. Mutations in the C-terminal domain of Sonic Hedgehog cause holoprosencephaly. *Hum. Mol. Genet.* **6**, 1847–1853 (1997).

[5] Nanni, L. et al. The mutational spectrum of the Sonic Hedgehog gene in holoprosencephaly: SHH mutations cause a significant proportion of autosomal dominant holoprosencephaly. *Hum. Mol. Genet.* **8**, 2479–2488 (1999).

[6] Sherry, S. et al. dbSNP: the NCBI database of genetic variation. *Nucleic Acids Res.* **29**, 308–311 (2001).

[7] Sondka, Z. et al. The COSMIC cancer gene census: describing genetic dysfunction across all human cancers. *Nat. Rev. Cancer* **18**, 696–705 (2018).

[8] Landrum, M. J. & Kattman, B. L. ClinVar at five years: Delivering on the promise. *Hum. Mutat.* **39**, 1623–1630 (2018).

[9] Wu, P. et al. Integration and analysis of CPTAC proteomics data in the context of cancer genomics in the cBioPortal. *Mol Cell Proteomics* **18**, 1893 (2019).

[10] Hung, J.-H. & Weng, Z. Sequence alignment and homology search with BLAST and ClustalW. *Cold Spring Harb Protoc* **2016**, pdb.prot093088 (2016).

[11] Drozdetskiy, A., Cole, C., Procter, J. & Barton, G. J. JPred4: a protein secondary structure prediction server. *Nucleic Acids Res.* **43**, W389–W394 (2015).

Supplementary Note 1

Plasmids used

Human Sonic Hedgehog and EGFP plasmids

Name	Source
hSHH	Origene RC222175
hSHH untagged	Origene RC222175, stop after hSHH(S462)
pEGFP-c1	Addgene vdb2487

Organelle marker protein plasmids

Addgene #	Plasmid	Origin of localization sequence	Organelle
55073	mCherry-Lysosomes-20	LAMP1	lysosomes
55052	mCherry-Golgi-7	β-galactosyltransferase	Golgi
85048	pmScarlet-Giantin-C1	Giantin	Golgi

Primers used

Deletions and truncations

Cloning method: Inverse PCR

Construct	Forward Primer	Reverse primer
hSHH(Δ369-391) (Δ1st helix)	GCTACGCGGTCATCCCCGCGCACGGA	GCGCGCGGGGATGACCGCAGAACCC
hSHH(Δ431-447) (Δ2nd helix)	TGCGGGGGCCACCGCGGACAGCGAGGCCCTGC	GCCTCGCTGTCCGCGGTGGCCCCCGCACCCGGA
hSHH(Δ393-424) (Δloop)	GCTGCACTGGCGCCCGGTGCGGGGG	GGTGGCCCCCGCACCGGGCGCCAGT
hSHH(E368*) (ΔSRR-Myc-DDK)	TCGTGCTACGCGGTCATCACGCGTACGCGGCCGCTCGAGCAGAA	CGAGCGGCCGCGTACGCGTGATGACCGCGTAGCACGAGGCCA

Single amino acid mutations

Cloning method: Site directed mutagenesis

Hint domain mutants

**Construct**	**Forward Primer**	**Reverse primer**
hSHH(C198A)	CAAATCGGGAGGCGCCTTCCCGGGCTCGG	CCGAGCCCGGGAAGGCGCCTCCCGATTTG
hSHH(D243A)	TTCCTCACTTTCCTGGCCCGCGACGACGGCGCC	GGCGCCGTCGTCGCGGGCCAGGAAAGTGAGGAA
hSHH(T267A)	CGCCTGCTGCTCGCCGCCGCGCACCTG	CAGGTGCGCGGCGGCGAGCAGCAGGCG
hSHH(H270A)	GCTCACCGCCGCGGCCCTGCTCTTTGTGG	CCACAAAGAGCAGGGCCGCGGCGGTGAGC
hSHH(C363A)	GGTGCTGGCCTCGGCC TACGCGGTCATCG	CGATGACCGCGTAGGCCGAGGCCAGCACC
hSHH(E368A)	GCGGTCATCGCGGAGCACAGCTGGGCGCACCGG	GCTGTGCTCCGCGATGACCGCGTAGCACGAGGC
hSHH(E368*)	GCTACGCGGTCATCTGAGAGCACAGCTGGG	CCCAGCTGTGCTCTCAGATGACCGCGTAGC

1st helix mutants

Construct	Forward Primer	Reverse primer
hSHH(W372A)	GAGGAGCACAGCGCGGCGCACCGGGC	GCCCGGTGCGCCGCGCTGTGCTCCTC
hSHH(H374A)	GTCATCGAGGAGCACAGCTGGGCGGCCCGGGCCTT	GCTGTGCTCCTCGATGACCGCGTAGCACGAGGCCAGCA
hSHH(R375A)	TGGGCGCACGCGGCCTTCGCGCCCTTCCGCCTG	CGCGAAGGCCGCGTGCGCCCAGCTGTGCTCCTC
hSHH(F377A)	CACCGGGCCGCCGCGCCCTTCCGCCTGGCGCAC	GAAGGGCGCGGCGGCCCGGTGCGCCCAGCTGTG
hSHH(P379A)	GCCTTCGCGGCCTTCCGCCTGGCGCACGCGCTC	CAGGCGGAAGGCCGCGAAGGCCCGGTGCGCCCA
hSHH(F380A)	GCCTTCGCGCCCGCCCGCCTGGCGCA	TGCGCCAGGCGGGCGGGCGCGAAGGC
hSHH(R381A)	TTCGCGCCCTTCGCCCTGGCGCACGCG	CGCGTGCGCCAGGGCGAAGGGCGCGAA
hSHH(L382A)	CCCTTCCGCGCGGCGCACGCGCTCCTGGCTGCA	CGCGTGCGCCGCGCGGAAGGGCGCGAAGGCCCG
hSHH(L382E)	TTCCGCGAAGCGCACGCGCTCCTGGCTGCACT	TGCGCTTCGCGGAAGGGCGCGAAGGCCCGGT
hSHH(H384A)	TTCCGCCTGGCGGCCGCGCTCCTGGC	GCCAGGAGCGCGGCCGCCAGGCGGAA
hSHH(L386A)	GCCTGGCGCACGCGGCCCTGGCTGCACTGGC	GCCAGTGCAGCCAGGGCCGCGTGCGCCAGGC
hSHH(L386E)	TTCCGCCTGGCGCACGCGGAACTGGCTGCACT	TGCGCCAGGCGGAAGGGCGCGAAGGCCCGGT
hSHH(L387A)	GCGCACGCGCTCGCGGCTGCACTGGC	GCCAGTGCAGCCGCGAGCGCGTGCGC
hSHH(L387E)	GCGCACGCGCTCGAGGCTGCACTGGC	GAGCGGCCGCGTACGCGTGCTGGACTTGACCGC
hSHH(L390A)	CGCTCCTGGCTGCAGCGGCGCCCGCGCGCAC	GTGCGCGCGGGCGCCGCTGCAGCCAGGAGCG
hSHH(L390E)	CACGCGCTCCTGGCTGCAGAAGCGCCCGCGCGCA	AGCCAGGAGCGCGTGCGCCAGGCGGAAGGGCGCGA
hSHH(L382A/L386	Sequential mutagenesis; L382A-L386A-L390A	

A/L390A)

2nd helix mutants

Construct	Forward Primer	Reverse primer
hSHH(I432A)	GGCCACCGCGGGCGCCCACTGGTACTCGCA	TGCGAGTACCAGTGGGCGCCCGCGGTGGCC
hSHH(H433A)	CACCGCGGGCATCGCCTGGTACTCGCAGC	GCTGCGAGTACCAGGCGATGCCCGCGGTG
hSHH(W434A)	CGCGGGCATCCACGCGTACTCGCAGCTGC	GCAGCTGCGAGTACGCGTGGATGCCCGCG
hSHH(Y435A)	GGGCATCCACTGGGCCTCGCAGCTGCTCT	AGAGCAGCTGCGAGGCCCAGTGGATGCCC
hSHH-Y435D	GCGGGCATCCACTGGGACTCGCAGCTGCTCTAC	GTAGAGCAGCTGCGAGTCCCAGTGGATGCCCGC
hSHH-Y435F	GCGGGCATCCACTGGTTCTCGCAGCTGCTCTAC	GTAGAGCAGCTGCGAGAACCAGTGGATGCCCGC
hSHH-Q437A	CATCCACTGGTACTCGGCGCTGCTCTACCAAATAG	CTATTTGGTAGAGCAGCGCCGAGTACCAGTGGATG
hSHH-L438A	CACTGGTACTCGCAGGCCCTCTACCAAATAGGC	GCCTATTTGGTAGAGGGCCTGCGAGTACCAGTG
hSHH-L439A	GTACTCGCAGCTGGCCTACCAAATAGGCACC	GGTGCCTATTTGGTAGGCCAGCTGCGAGTAC
hSHH-Y440A	CTCGCAGCTGCTCGCCCAAATAGGCACCT	AGGTGCCTATTTGGGCGAGCAGCTGCGAG
hSHH-Q441A	CCACTGGTACTCGCAGCTGCTCTACGCAATAGGC	CGCTGTCCAGGAGCCAGGTGCCTATTGCGTAGAG
hSHH-I442A	CTGGTACTCGCAGCTGCTCTACCAAGCAGGCACC	CCTCGCTGTCCAGGAGCCAGGTGCCTGCTTGGTA
hSHH(G443A)	CAAATAGCCACCTGGCTCCTGGACAGCGAGGCCC	CCAGGTGGCTATTTGGTAGAGCAGCTGCGAGTAC
hSHH-T444A	CTCTACCAAATAGGCGCCTGGCTCCTGGACAGC	GCTGTCCAGGAGCCAGGCGCCTATTTGGTAGAG
hSHH-W445A	TACCAAATAGGCACCGCCCTCCTGGACAGCGAG	CTCGCTGTCCAGGAGGGCGGTGCCTATTTGGTA

hSHH-L446A	CAAATAGGCACCTGGGCCCTGGACAGCGAGGCC	GGCCTCGCTGTCCAGGGCCCAGGTGCCTATTTG
hSHH-L447A	ATAGGCACCTGGCTCGCGGACAGCGAGGCCCTG	CAGGGCCTCGCTGTCCGCGAGCCAGGTGCCTAT
hSHH(D448A)	CACCTGGCTCCTGGCCAGCGAGGCCCTGCA	TGCAGGGCCTCGCTGGCCAGGAGCCAGGTG

Primers to remove stop codons

hSHH-Unstop-1	TCCAGCACGCGTACGCGGCCGCTCGAGCAGAAAC	CGTACGCGTGCTGGACTTGACCGCCATGCCCAGC
hSHH-Unstop-2	CGGTCAAGTCCAGCACGCGTACGCGGCCGCT	AGCGGCCGCGTACGCGTGCTGGACTTGACCG

EGFP fusion proteins

Cloning method: Gibson Assembly

	**Template**	**Forward primer**	**Reverse primer**
**EGFP-SRR**			
Insert	pEGFP-c1	GATCTGCCGCCGCGATCGCCATGGTGAGCAAGGGCGAG	CTGTGCTCCTCGATGACCGCAGAACCCCCACCACCGCTGCCACCACCGCCCTTGTACAGCTCGTCCATGC
Backbone	pCMV6-hSHH untagged	GCGGTCATCGAGGAGCACAGCTGGGCGCAC	GGCGATCGCGGCGGCAGATCTCCTCGGTAC
**EGFP-Hint(C198A)**			
Insert	pCMV6-hSHH(C198A)	GCAGCGGTGGTGGGGGTTCTTCGGTGAAAGCAGAGAACTCGGTG	GCCTCGTGCTACGCGGTCATCTGACGTACGCGGCCGCTC

	untagged		
Backbone	EGFP-SRR	TGACGTACGCGGCCGCTCGAGCAGAAACTCAT	AGAACCCCCACCACCGCTGCCACCACC
**EGFP-Hint(C198A)-SRR**			
Insert	pCMV6-hSHH(C198A) untagged	GCAGCGGTGGTGGGGGTTCTTCGGTGAAAGCAGAGAACTCGGTG	GAGCGGCCGCGTACGTCAGCTGGACTTGACCGCCATGCC
Backbone	EGFP-SRR	TGACGTACGCGGCCGCTCGAGCAGAAACTCAT	AGAACCCCCACCACCGCTGCCACCACC
