# Supplementary material for: Dual roles of the sterol recognition region in Hedgehog protein modification
Source: Commun Biol. 2020 May 21;3:250. doi: 10.1038/s42003-020-0977-2 (PMC7242414; doi:10.1038/s42003-020-0977-2)
Supplement: Supplementary file 2 — Description of Additional Supplementary Files [file 42003_2020_977_MOESM2_ESM.pdf]

Description of Additional Supplementary Files

File Name: Supplementary Data 1

Description: Model of the human Sonic Hedgehog sequence (NP_000184.1, residues 363-462) generated using the Ab Initio Rosetta fragment assembly method on the online Robetta server (http://robetta.bakerlab.org/).
