# Supplementary material for: Dual roles of the sterol recognition region in Hedgehog protein modification
Source: Commun Biol. 2020 May 21;3:250. doi: 10.1038/s42003-020-0977-2 (PMC7242414; doi:10.1038/s42003-020-0977-2)
Supplement: Supplementary file 4 — Reporting summary [file 42003_2020_977_MOESM4_ESM.pdf]

nature research

Corresponding author(s): Alison E. Ondrus

Last updated by author(s): Apr 19, 2020

Reporting Summary

Nature Research wishes to improve the reproducibility of the work that we publish. This form provides structure for consistency and transparency in reporting. For further information on Nature Research policies, see Authors & Referees and the Editorial Policy Checklist .

Statistics

For all statistical analyses, confirm that the following items are present in the figure legend, table legend, main text, or Methods section.

n/a	Confirmed	
☐	☒	The exact sample size (*n*) for each experimental group/condition, given as a discrete number and unit of measurement
☐	☒	A statement on whether measurements were taken from distinct samples or whether the same sample was measured repeatedly
☒	☐	The statistical test(s) used AND whether they are one- or two-sided<br>*Only common tests should be described solely by name; describe more complex techniques in the Methods section.*
☒	☐	A description of all covariates tested
☒	☐	A description of any assumptions or corrections, such as tests of normality and adjustment for multiple comparisons
☐	☒	A full description of the statistical parameters including central tendency (e.g. means) or other basic estimates (e.g. regression coefficient) AND variation (e.g. standard deviation) or associated estimates of uncertainty (e.g. confidence intervals)
☒	☐	For null hypothesis testing, the test statistic (e.g. *F*, *t*, *r*) with confidence intervals, effect sizes, degrees of freedom and *P* value noted<br>*Give P values as exact values whenever suitable.*
☒	☐	For Bayesian analysis, information on the choice of priors and Markov chain Monte Carlo settings
☒	☐	For hierarchical and complex designs, identification of the appropriate level for tests and full reporting of outcomes
☐	☒	Estimates of effect sizes (e.g. Cohen's *d*, Pearson's *r*), indicating how they were calculated

Our web collection on statistics for biologists contains articles on many of the points above.

Software and code

Policy information about availability of computer code

Data collection	No commercial or open-source code was used to collect data for this study
Data analysis	GraphPad Prism, Bio-Rad Image Lab Software v6.0 (https://www.bio-rad.com/en-us/product/image-lab-software), PROMALS3D (http://prodata.swmed.edu/promals3d/promals3d.php), JPred (http://www.compbio.dundee.ac.uk/jpred/), HELIQUEST (https://heliquest.ipmc.cnrs.fr/), Robetta (http://new.robetta.org/), MCPep (http://bental.tau.ac.il/MCPep/), ImageJ Coloc2 (https://imagej.net/Coloc_2). All references are provided in the manuscript.

For manuscripts utilizing custom algorithms or software that are central to the research but not yet described in published literature, software must be made available to editors/reviewers. We strongly encourage code deposition in a community repository (e.g. GitHub). See the Nature Research guidelines for submitting code & software for further information.

Data

Policy information about availability of data

All manuscripts must include a data availability statement. This statement should provide the following information, where applicable:

- Accession codes, unique identifiers, or web links for publicly available datasets
- A list of figures that have associated raw data
- A description of any restrictions on data availability

The datasets generated during and/or analyzed during the current study are available from the corresponding author on reasonable request.

Field-specific reporting

Please select the one below that is the best fit for your research. If you are not sure, read the appropriate sections before making your selection.

Life sciences study design

All studies must disclose on these points even when the disclosure is negative.

Sample size	All datapoints represent the average of three or more biological replicates ± s.d.
Data exclusions	No data was excluded from the analysis
Replication	All attempts at replication were successful
Randomization	Randomization was not applied; internal controls were included in each experiment
Blinding	Blinding was not performed

Reporting for specific materials, systems and methods

We require information from authors about some types of materials, experimental systems and methods used in many studies. Here, indicate whether each material, system or method listed is relevant to your study. If you are not sure if a list item applies to your research, read the appropriate section before selecting a response.

Materials & experimental systems

n/a	Involved in the study	
☐	☒	Antibodies
☐	☒	Eukaryotic cell lines
☒	☐	Palaeontology
☒	☐	Animals and other organisms
☒	☐	Human research participants
☒	☐	Clinical data

Methods

n/a	Involved in the study	
☒	☐	ChIP-seq
☒	☐	Flow cytometry
☒	☐	MRI-based neuroimaging

Antibodies

Antibodies used	Primary antibodies: Shh Antibody (E-1) (N-terminus), (Santa Cruz Biotechnology, sc-365112), Sonic Hedgehog/Shh C-Terminus Antibody (R&D Systems, AF445), Loading control: Karyopherin β1 (H-7) (Santa Cruz Biotechnology, sc-137016). Secondary antibodies: Donkey anti-Goat IgG (H+L) Cross-Adsorbed Secondary Antibody, Alexa Fluor 647 (Invitrogen, A-21447), Donkey anti-Mouse IgG (H+L) Cross-Adsorbed Secondary Antibody, Alexa Fluor 488 (Invitrogen, A-21202).
Validation	All antibodies used were validated for Western Blot applications in images provided by the vendor and peer-reviewed citations

Eukaryotic cell lines

Policy information about cell lines

Cell line source(s)	HEK293T (ATCC, CRL-3216)
Authentication	Cells were obtained from ATCC and expanded directly from the original vial
Mycoplasma contamination	Cell lines tested negative for mycoplasma contamination, checked monthly with the MycoAlert Mycoplasma Detection Kit (Lonza, LT07-118)
Commonly misidentified lines (See ICLAC register)	N/A
